# Microplastic Contamination in Industrially Packaged and Locally Produced Ice Creams: Occurrence, Characteristics, Exposure Assessment, and Pollution Risk

**DOI:** 10.3390/foods15142517

**Published:** 2026-07-16

**Authors:** Tanju Mutlu, Yusuf Ceylan, Barış Karslı

**Affiliations:** 1 Vocational School of Technical Sciences, Recep Tayyip Erdoğan University, 53100 Rize, Türkiye; tanju.mutlu@erdogan.edu.tr; 2 Faculty of Fisheries, Recep Tayyip Erdoğan University, 53100 Rize, Türkiye; yusuf.ceylan@erdogan.edu.tr

**Keywords:** microplastics, ice cream, dietary exposure, food safety, food-contact materials

## Abstract

Microplastic (MP) contamination in foods has emerged as an increasing food safety concern; however, information regarding ice cream products remains limited. This study comparatively investigated MP contamination in locally produced unpackaged and industrially packaged ice creams marketed in Türkiye. A total of 24 samples (19 industrially packaged and 5 locally produced unpackaged) were analyzed using microscopic examination followed by ATR-FTIR polymer verification. Detected MPs were characterized according to polymer type, morphology, size, and color. MPs were detected in 100% of locally produced unpackaged samples and 42.1% of industrially packaged samples. EVA and ABS–EVA were the predominant polymer types, whereas fibers and black particles were the dominant morphology and color, respectively. A polymer-weighted pollution risk index (pRi) and a deterministic exposure assessment were also applied. Both the mean pRi values and estimated daily intake (EDI) were higher in locally produced unpackaged ice creams than in industrially packaged products. These findings suggest that differences in handling practices, environmental exposure, and food-contact materials may influence MP contamination. Overall, the results indicate that ice cream may represent a potential source of dietary MP exposure and highlight the importance of implementing effective contamination-control measures throughout production, packaging, and retail handling. This study provides valuable comparative baseline data for future food safety and dietary exposure assessments.

## 1. Introduction

Microplastics (MPs) are persistent pollutants smaller than five millimeters that originate from the fragmentation of larger plastic materials through physical, chemical, and biological processes and are now widely distributed throughout the environment [1,2]. Increasing evidence indicates that plastics used in food packaging and processing can fragment into MPs, raising concerns not only about environmental pollution but also about food safety and human health [3,4,5]. Accordingly, MPs have been detected in a wide variety of foods, including seafood, honey, dairy products, processed foods, and snacks, highlighting food as an important route of human exposure [6,7,8,9,10,11,12].

Food packaging materials, particularly polyethylene (PE), polypropylene (PP), polystyrene (PS), and polyethylene terephthalate (PET), may release MPs into foods as a result of mechanical stress, temperature fluctuations, prolonged storage, and food contact [9,13,14]. Previous studies have reported MP contamination in packaged dairy products, such as milk and cheese, and demonstrated that polymers originating from packaging materials can migrate into food matrices [11,15]. Recent reviews have further emphasized that food packaging is one of the major contributors to dietary MP exposure [16]. In addition to acting as physical contaminants, MPs may also serve as carriers of chemical pollutants, including phthalates, heavy metals, pesticides, and other toxic compounds associated with adverse biological effects [5,14,17].

Although the potential health effects of MPs remain incompletely understood, experimental and epidemiological studies suggest possible associations with oxidative stress, inflammation, metabolic disturbances, neurotoxicity, and reproductive toxicity [18,19,20]. Therefore, investigating the occurrence, characteristics, and potential dietary exposure of MPs in different food products remains essential for improving food safety risk assessment.

Ice cream is a widely consumed frozen dairy product, particularly among children and adolescents, and therefore represents a potentially important yet understudied source of dietary MP exposure. Despite multiple opportunities for contamination during production, packaging, storage, transportation, and retail handling, only a limited number of studies have directly investigated MPs in ice cream. Zhang et al. [21] reported the occurrence of various polymer types and particle morphologies in commercially packaged ice creams marketed in China. Similarly, Akkemik et al. [22] detected MPs in commercially available ice creams in Türkiye and suggested that contamination may originate from multiple stages throughout the production and packaging chain. However, comparative information regarding industrially packaged and locally produced unpackaged ice creams remains scarce, and the influence of open handling practices, environmental exposure, and food-contact materials on contamination patterns has not yet been adequately explored.

To the best of our knowledge, the present study is among the first to comparatively investigate microplastic contamination in industrially packaged and locally produced unpackaged ice cream products. In addition to determining MP abundance, size, morphology, and polymer composition, this study evaluates contamination patterns associated with different production and handling conditions. Unlike previous studies that focused primarily on packaged industrial products, the inclusion of unpackaged locally produced ice creams provides additional insight into the potential influence of open handling and environmental exposure on MP contamination. Furthermore, a polymer-weighted pollution risk index (pRi) was applied as a comparative indicator of contamination, together with a deterministic exposure assessment, to provide a preliminary evaluation of potential consumer exposure associated with ice cream consumption. By addressing these knowledge gaps, this study provides comparative baseline data on MP contamination in packaged and unpackaged ice cream products and contributes to a better understanding of potential contamination pathways and consumer exposure associated with frozen dairy products.

## 2. Material and Method

### 2.1. Sampling and Sample Preparation

A total of 24 ice cream samples were collected to investigate the presence of microplastics (MPs), including 19 industrially packaged products and 5 unpackaged products obtained from local producers (Table 1). The samples were obtained from local markets and manufacturers’ sales outlets. Samples were selected to represent both industrially packaged and locally produced unpackaged ice cream products, ensuring that packaged products remained unopened until sampling. Locally produced unpackaged ice creams were sampled directly from stainless-steel containers displayed in refrigerated retail cabinets. Samples were transferred into pre-cleaned glass containers with aluminum foil-lined lids immediately after purchase. Each commercially available ice cream product was considered an independent biological sample. Therefore, biological replicates were not included in the study design, as the aim was to evaluate MP contamination among individual retail products rather than to assess within-product variability. All samples were transported to the laboratory under contamination-controlled conditions while maintaining the cold chain to minimize potential cross-contamination. Upon arrival, samples were transferred into pre-cleaned glass beakers and covered with aluminum foil. For the digestion of organic matter, 30% hydrogen peroxide (H_2_O_2_) was added at an approximate sample ratio of 1:10 depending on the organic content of the samples. The relatively high oxidant volume was selected to facilitate digestion of the protein- and lipid-rich dairy matrix. The beakers were then sealed and placed in a shaking water bath at 65 °C and 80 rpm for approximately 72 h. Following digestion, the resulting solution was filtered through 8 µm pore-size PCTE membrane filters using a vacuum filtration system. The filters were subsequently transferred to glass Petri dishes, dried at room temperature, and stored for microscopic analysis [23]. The retained particles were visually examined using a Leica DM500 compound light microscope (Leica Microsystems, Germany) at 10× and 40× magnifications. Digital micrographs were recorded, and particle dimensions were measured using ImageJ software (Version 1.54d, National Institutes of Health, Bethesda, MD, USA). Morphological classification followed the criteria proposed by Hidalgo-Ruz et al. [23], whereby fibers were defined as elongated, thread-like particles with a relatively uniform thickness and a length substantially greater than their width, whereas fragments were identified as irregularly shaped particles with angular or uneven edges. Representative microscopic images of the identified particle morphologies together with their corresponding ATR-FTIR spectra are provided in Figure S1.

All laboratory procedures were conducted in a controlled clean-room environment to minimize contamination. Cotton laboratory coats and polymer-free gloves were used throughout the experiments. Additionally, all liquids (distilled water and H_2_O_2_) were pre-filtered using 47 mm diameter filter papers with a pore size of 1.2 µm. All laboratory equipment (including beakers, filter units, conical flasks, aspirator bottles, and Petri dishes) was thoroughly rinsed with filtered water and covered with aluminum foil prior to use.

Procedural blanks and airborne contamination controls were included during all analytical batches. Particles detected in procedural blanks were compared with sample-derived particles based on morphology, color, and polymer type. When matching characteristics were observed, the corresponding particles were excluded from the final dataset. The results of the procedural blank analyses and the ATR-FTIR classification of all visually suspected particles are provided in Supplementary Tables S1 and S2, respectively. Although extensive quality-control measures were implemented, the possibility of airborne fiber contamination cannot be completely excluded, particularly because fiber-shaped particles predominated among the detected MPs.

### 2.2. Polymer Detection and Verification

Determination and verification of the polymer type of suspected microplastic particles were performed using ATR FT-IR (Attenuated Total Reflection Fourier Transform Infrared Spectroscopy). Particle spectra were acquired over the range of 4000–650 cm^−1^ at a spectral resolution of 2 cm^−1^ using four scans. The resulting spectra were compared with both the Spectrum Search Plus Software library (Version 6.3.0, PerkinElmer Inc., Waltham, MA, USA) and reference polymer spectra. Because particle size represents an important limitation of ATR-FTIR analysis, a stereo microscope or a 10× table-top magnifier was used to place small particles on the ATR crystal, allowing particles as small as 100 µm to be analyzed successfully [24,25,26]. Polymer identification was accepted only when the spectral library match score was ≥70% and the main diagnostic absorption bands were manually verified. Spectra with poor signal quality, low signal-to-noise ratios, or ambiguous matches were excluded from the dataset. The ATR crystal was cleaned with 70% ethanol and a lint-free tissue between successive measurements to prevent spectral carryover. Four scans were selected because they provided spectra of sufficient quality for reliable polymer identification while maintaining analytical efficiency. A total of 38 visually suspected particles were subjected to ATR-FTIR verification. Of these, 21 particles were confirmed as microplastics and included in the final dataset, whereas 13 were identified as cellulose-based particles, 2 as hair, and 2 yielded ambiguous spectra that did not meet the predefined identification criteria (Table S2). Only ATR-FTIR-confirmed microplastic particles were included in the subsequent analyses.

### 2.3. Quality Control

To minimize MP contamination and to ensure the reliability of analytical results, comprehensive quality control measures were implemented throughout the study. All procedures were conducted in an isolated laboratory dedicated exclusively to MP analyses, without ventilation or air-conditioning systems. Access to the laboratory was restricted to reduce the risk of airborne contamination. To limit external contamination, 100% cotton laboratory coats and nitrile gloves were worn at all times. All equipment was cleaned with 70% ethanol prior to each analysis and subsequently rinsed with filtered or distilled water. Throughout the study, only glass or metal materials were used, and all glassware and instruments were pre-cleaned with filtered water before use. Samples were processed in glass containers and protected during preparation steps by covering them with watch glasses or aluminum foil. Filtration procedures were carried out under a laminar flow cabinet, and filters were stored in glass Petri dishes until analysis. Three procedural blanks containing only filtered water were processed alongside the samples following the same analytical procedures. Only one green fiber was detected in a single procedural blank and was identified as polyester by ATR-FTIR analysis, whereas no particles were observed in the remaining two blanks. The corresponding particle was excluded from the final dataset. Detailed results of the procedural blank analyses are presented in Table S1. In addition, control filters were placed in the working area to monitor airborne contamination, and particles similar to those detected in negative controls were excluded from the evaluation.

### 2.4. Estimated Daily Intake (EDI) of Microplastics

A deterministic exposure assessment was applied to estimate potential dietary exposure to microplastics through ice cream consumption. The estimated daily intake (EDI) was calculated using Equation (1):

EDI = (Ma × Mc)/BW(1)

where EDI represents the estimated daily intake of microplastics (particles kg body weight^−1^ day^−1^), Ma is the average daily ice cream consumption rate (g day^−1^), Mc is the concentration of microplastics detected in the analyzed samples (particles g^−1^), and BW is the average body weight (kg). The average daily ice cream consumption value (12.6 g day^−1^) and mean body weight (73.7 kg) were adopted from nationally representative dietary and demographic data previously reported for the Turkish population [22]. EDI values were calculated separately for industrially packaged and locally produced unpackaged ice cream samples in order to evaluate potential differences in consumer exposure associated with product type. For industrially packaged products, the EDI range reflects a uniform microplastic concentration across all positive samples; therefore, minimum and maximum values are identical. Because age-specific national ice cream consumption data were unavailable, nationally representative mean daily ice cream consumption (12.6 g day^−1^) and average body weight (73.7 kg) values for the general Turkish population were used to provide a preliminary deterministic exposure estimate.

### 2.5. The Pollution Risk Index

The Pollution Risk Index (pRi) was used in the study to assess the potential risk of microplastics. pRi was calculated by summing the toxicity coefficient (Tni) assigned to each individual microplastic particle detected in a sample, based on its polymer type [27]. The total risk score for each sample was defined as follows:

(2) $$pRi=\sum_{i=1}^{n}Tn_{i}$$

The calculated pRi values were used as a relative indicator to compare contamination levels among samples and producer types. Because no universally accepted classification criteria are currently available for interpreting pRi values in food matrices, the present study does not apply categorical pollution levels or toxicological risk thresholds.

Compared with simple particle counting, this approach provides a more comprehensive assessment because particles composed of polymers with higher toxicity coefficients contribute proportionally greater values to the overall pRi, while the cumulative score also reflects the overall contamination burden of each sample.

### 2.6. Statistical Analysis

All analyses were conducted using R Studio (4.0) [28]. The dplyr package was used for data cleaning, editing, and calculations; ggplot2 for graphical visualization; tidyr for frequency table and categorical variable analyses; and stats for statistical tests [29,30]. Differences in microplastic occurrence and polymer distributions were evaluated using Fisher’s exact test. Particle size was compared among producer types and polymer types using the Wilcoxon rank-sum and Kruskal–Wallis tests, respectively.

## 3. Results

### 3.1. Occurrence, Distribution, and Characteristics of Microplastics in Ice Cream Samples

In this study, a total of 24 ice cream samples, including locally produced unpackaged and industrially packaged products obtained from retail outlets in Türkiye, were evaluated for MP contamination. Microplastics were characterized according to polymer type, particle size, morphology, and color. The results revealed statistically significant differences in both the physical characteristics of the detected MPs and the level of contamination between the two producer types. Representative microscopic images illustrating the identified MP morphologies (fiber and fragment) together with their corresponding ATR-FTIR spectra are provided in Figure S1.

**Figure 1:**
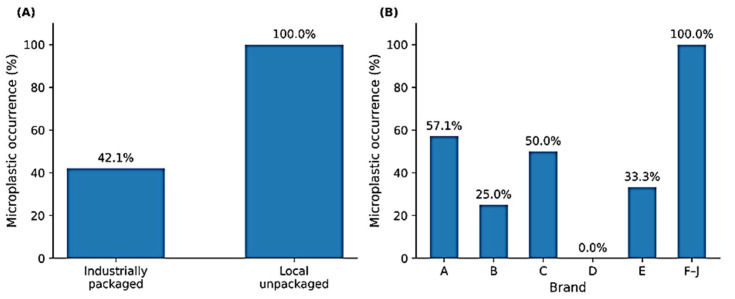
Detection frequency of microplastics in ice cream samples. (**A**) Occurrence according to producer type. (**B**) Occurrence according to brand categories.

MPs were detected in 100% of the samples obtained from local manufacturers. In contrast, MPs were detected in 42.1% of the samples from industrially packaged products. The detection frequency differed significantly between the two producer types (*p* < 0.05). These findings indicate that MP contamination was significantly more frequent in locally produced unpackaged ice creams than in industrially packaged products within the analyzed dataset (Figure 1).

**Figure 2:**
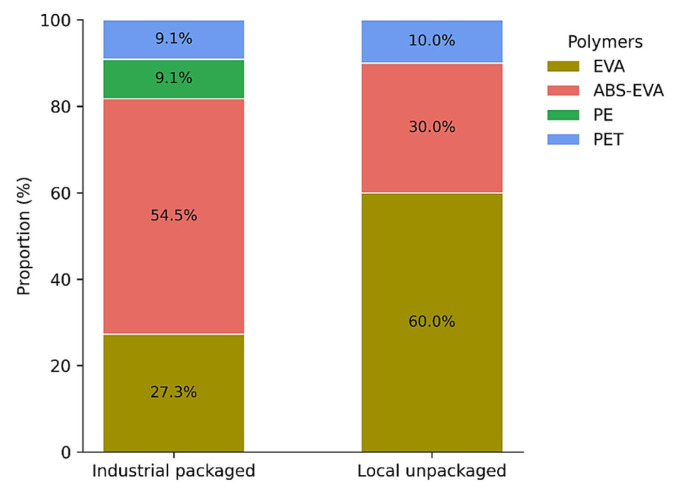
Polymer composition (%) of microplastics detected in industrially packaged and locally produced unpackaged ice cream samples.

Microplastics were detected in all locally produced ice cream samples, with ethylene-vinyl acetate (EVA) accounting for 60.0% and ABS-EVA for 30.0% of the identified polymer types, indicating that EVA- and ABS-EVA-based particles represented the dominant polymer categories detected in locally produced samples. Among the contaminated industrially packaged products, ABS-EVA (54.5%) and EVA (27.3%) were the most prevalent polymer types (Figure 2). A statistically significant difference was observed in the polymer distribution between locally produced and industrially packaged ice cream samples (*p* < 0.05). Packaging materials of industrially packaged products consisted primarily of polypropylene (PP), while one product was packaged in biaxially oriented polypropylene (BOPP) (Table 1). However, the identified MP polymers differed from the declared packaging materials, with EVA, ABS-EVA, PET, and PE representing the detected polymer types in contaminated samples.

**Figure 3:**
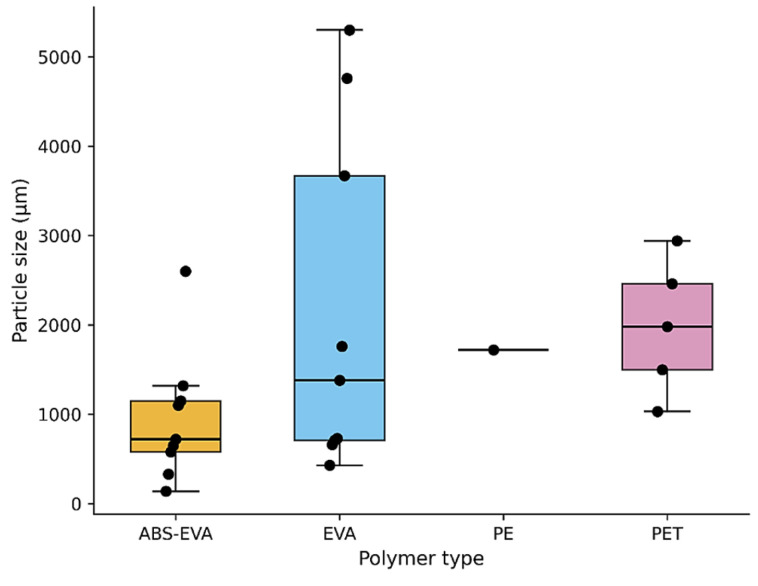
Distribution of microplastic particle sizes according to polymer type. Boxes represent the interquartile range (IQR), horizontal lines indicate the median, whiskers represent the data range, and dots correspond to individual particles.

The overall median particle size was 1099 µm, with particle sizes ranging from 138 to 5300 µm. Microplastic particle sizes varied across polymer types (Figure 3). ABS-EVA particles exhibited a relatively concentrated size distribution, primarily within the 500–1500 µm range, whereas EVA particles showed a broader distribution, encompassing both smaller and larger size fractions. PET microplastics were mainly observed within the 1500–3000 µm range, while polyethylene (PE) particles showed a relatively uniform size distribution. Despite these apparent differences, statistical analysis revealed no significant variation in microplastic size distributions either between producer types or among polymer categories (*p* > 0.05).

**Figure 4:**
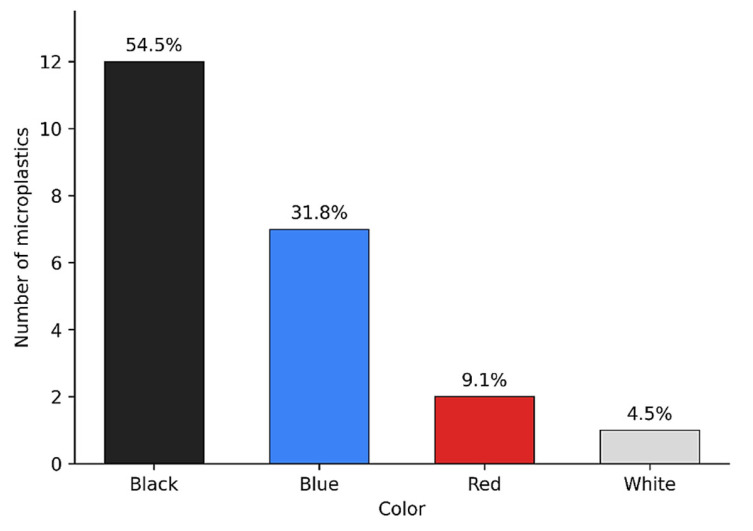
Color distribution (%) of detected microplastics in ice cream samples.

Black particles accounted for the highest proportion of all detected MPs, followed by blue, red, and white particles, respectively (Figure 4). Similar distribution patterns were observed in both locally produced and industrially packaged ice cream samples. Statistical analysis indicated no significant differences in microplastic color categories between the two producer types (*p* > 0.05). However, comparisons among color categories within the overall dataset showed statistically significant differences in their occurrence frequencies (*p* < 0.05).

**Figure 5:**
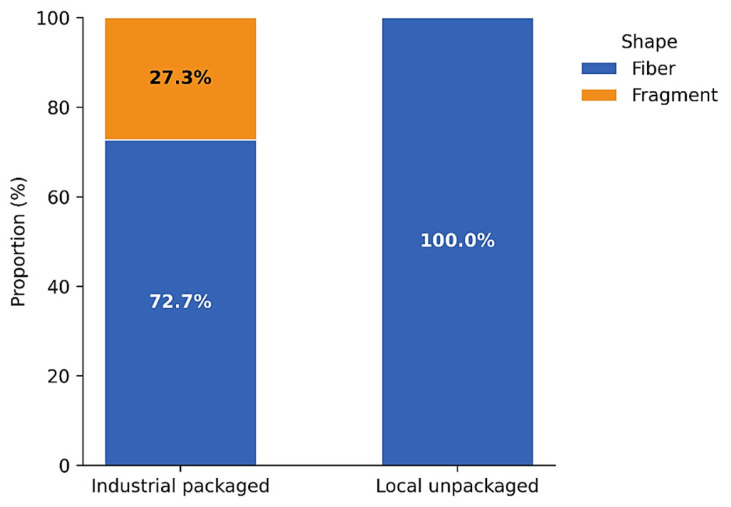
Shape distribution (%) of detected microplastic according to produces type.

Heatmap analysis revealed clear differences in microplastic morphology between producer types (Figure 5). In locally produced unpackaged ice creams, all detected microplastics were classified as fibers, whereas no fragments were observed. In contrast, both fibers and fragments were detected in industrially packaged products, with fibers representing the dominant morphological category. These findings suggest that microplastic morphology differed between producer types, with unpackaged products exhibiting a more uniform morphology and industrially packaged products showing greater morphological diversity.

**Figure 6:**
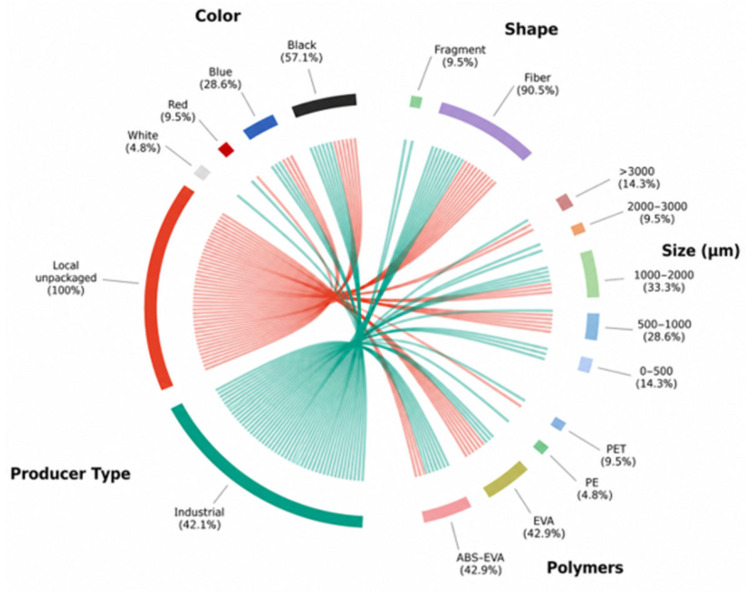
Chord diagram visualizing the relationships among sample group, polymer type, particle shape, color, and size class of microplastics detected in ice cream samples.

The chord diagram further illustrates that microplastic contamination is not driven by a single factor but rather reflects complex interactions among polymer type, morphology, size, and color (Figure 6). In particular, industrially packaged samples exhibited a more heterogeneous distribution pattern, suggesting more heterogeneous contamination patterns than those observed in locally produced samples.

### 3.2. Risk Assessment

**Figure 7:**
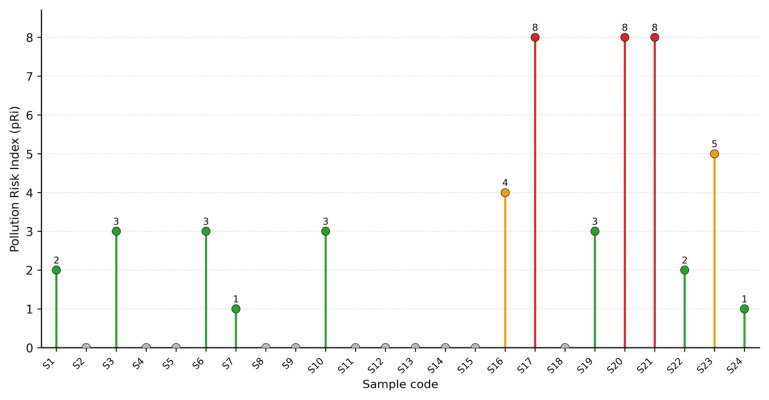
Pollution risk index of microplastic contamination in ice cream samples.

The presence of microplastics in ice cream samples was quantitatively evaluated using the pollution risk index (pRi) approach (Figure 7). The calculated pRi values varied among the analyzed samples, reflecting differences in the abundance and polymer composition of MPs. The highest pRi values were observed for samples D17 (Industrial), D20, and D21 (Local), whereas no MPs were detected in samples D2, D4, D5, D8, D9, D11–D15, and D18, resulting in pRi values of zero. Average pRi values were also calculated for each producer type. The mean pRi value was 5.75 for locally produced unpackaged samples and 1.42 for industrially packaged products. Although the difference between the two groups was not statistically significant (*p* > 0.05), the higher mean pRi observed in locally produced samples suggests a tendency toward greater relative MP contamination. Because pRi represents a comparative indicator of contamination rather than a direct measure of toxicological risk, these findings should be interpreted as reflecting relative differences in contamination levels among the analyzed samples. Furthermore, the occurrence of multiple polymer types in samples with elevated pRi values highlights the need for further investigations into the potential implications of mixed-polymer contamination in food products.

The estimated daily intake (EDI) values associated with ice cream consumption are summarized in Table 2. EDI calculations were performed using an average daily ice cream consumption of 12.6 g and an average body weight of 73.7 kg. The estimated exposure levels differed according to producer type. For industrially packaged ice cream products, the mean EDI value was calculated as 0.17 particles kg BW^−1^ day^−1^. In contrast, locally produced unpackaged ice creams exhibited a higher mean EDI value of 0.36 particles kg BW^−1^ day^−1^. Furthermore, the maximum EDI observed among locally produced samples reached 0.68 particles kg BW^−1^ day^−1^. The estimated annual exposure values were calculated as 62.05 and 131.40 particles kg BW^−1^ year^−1^ for industrially packaged and locally produced products, respectively. Under the maximum exposure scenario, annual exposure for locally produced samples reached 248.20 particles kg BW^−1^ year^−1^. The mean EDI estimated for locally produced unpackaged ice creams was approximately 2.1-fold higher than that estimated for industrially packaged products. Overall, consumers of locally produced unpackaged ice cream were estimated to experience higher microplastic exposure levels than consumers of industrially packaged products. This observation is consistent with the higher MP occurrence frequency observed in locally produced samples and may reflect differences in handling, environmental exposure, and food-contact conditions between product types. Because age-specific national ice cream consumption data are currently unavailable in Türkiye, the present exposure assessment was based on nationally representative mean daily consumption and body weight values for the general population. Consequently, the reported EDI values should be regarded as preliminary estimates and may underestimate exposure in children and other high-consumption groups, who generally consume greater amounts of ice cream relative to their body weight. Future studies incorporating age-specific consumption data and probabilistic exposure models would provide more refined estimates of dietary microplastic exposure.

## 4. Discussion

The detection of microplastics in both locally produced unpackaged and industrially packaged ice cream products suggests that frozen dairy products may contribute to dietary microplastic exposure and highlights the need for further investigation of contamination sources throughout the production and distribution chain.

### 4.1. Comparative Occurrence and Characteristics of MPs in Ice Cream Products

To date, studies specifically investigating microplastic contamination in ice cream products remain limited. Zhang et al. [21] reported the presence of microplastics in commercially packaged ice creams marketed in China, while Akkemik et al. [22]. detected microplastics in all analyzed ice cream samples collected in Türkiye. In the study of Akkemik et al. [22], filament-shaped particles represented the dominant morphology and several polymer types, including PP, PET, PE, and PS, were identified. Consistent with their findings, fiber-type particles were also dominant in the present study. However, unlike Akkemik et al. [22], the present study comparatively evaluated both industrially packaged and locally produced unpackaged ice creams. When evaluated proportionally, the detection of MPs in all locally produced unpackaged samples but only in a portion of industrially packaged products suggests that contamination patterns can differ depending on production, packaging, and handling conditions. Nevertheless, methodological differences, sample size limitations, and analytical variability between studies should also be considered when interpreting these findings. In the study of Akkemik et al. [22], MPs were detected in all analyzed samples regardless of brand type, suggesting that contamination may occur throughout multiple stages of production and distribution. Similarly, the detection of MPs in both producer groups in the present study may indicate that contamination may originate from different stages within the production and retail chain rather than from a single contamination source. In addition to differences between producer types, MP occurrence varied among the evaluated brands (Figure 1B). However, because most brands were represented by only a single sample, no meaningful statistical or comparative interpretation at the brand level can be made. Therefore, the observed differences among brands are presented solely as descriptive observations and should not be interpreted as evidence of differences among manufacturers or production practices.

Packaging materials are widely recognized as one of the potential contributors to food-borne microplastic contamination due to repeated contact between polymer-based materials and food matrices during processing, storage, and distribution [31,32]. Previous studies have reported that abrasion, fragmentation, and degradation of plastic packaging materials may contribute to the transfer of polymer particles into foods [33,34]. Similarly, Garrido Gamarro and Costanzo [35] emphasized that food processing environments, packaging systems, and handling conditions collectively contribute to MP contamination in processed foods. In the present study, industrially packaged products exhibited relatively broader polymer diversity, including PE and PET, which are commonly associated with food-contact materials and industrial packaging systems. Akkemik et al. [22] also reported the presence of PE, PET, PP, and PS polymers in commercially packaged ice cream products marketed in Türkiye and suggested that these polymers may originate from packaging materials, processing systems, or environmental contamination during production and storage stages. None of the detected polymer types directly matched the declared packaging materials (PP and BOPP) used in the industrially packaged products. These findings suggest that packaging alone may not fully explain the detected contamination patterns and that additional sources associated with processing, handling, or environmental exposure may also contribute to MP occurrence.

Although EVA and ABS-EVA polymers were more frequently detected in locally produced samples, the present study did not directly analyze packaging materials, production equipment, or environmental sources. Therefore, association between the detected polymers and specific contamination pathways should be considered hypothetical. The observed polymer distribution may reflect differences in handling conditions, environmental exposure, or food-contact materials; however, definitive source attribution cannot be established. Similar observations were reported by Buyukunal et al. [36] who emphasized that contamination profiles detected in food products may result from multiple overlapping contamination pathways rather than a single identifiable source. Therefore, the predominance of EVA and ABS-EVA polymers in locally produced unpackaged products may be associated with repeated handling processes, environmental exposure, or abrasion of food-contact materials during retail sale.

Although no statistically significant differences were observed in microplastic size distributions between producer types or polymer structures, the wide size ranges detected across all samples indicate that fragmentation processes may occur independently of production system. Similar patterns have been reported in dairy products such as milk and cheese, where microplastic size distributions were associated with cumulative abrasion, mechanical stress, and material aging during processing and handling stages [11]. Akkemik et al. [22] similarly reported a broad size distribution of particles in ice cream samples and suggested that repeated physical fragmentation during production, transportation, and storage may contribute to particle heterogeneity. Therefore, the absence of significant size-related differences in the present study may suggest that microplastic size characteristics reflect generalized physical degradation processes acting throughout the food production chain rather than specific contamination sources.

In the present study, black microplastics represented one of the most frequently detected color categories across the analyzed samples. Similar observations have previously been reported in dairy matrices, where dark-colored particles frequently dominate detected microplastic profiles [11]. Akkemik et al. [22] also reported the predominance of dark-colored particles in commercially packaged ice cream products. The authors suggested that particle pigmentation may be associated with additives, processing materials, or environmental exposure during production and storage stages. Similarly, the predominance of black particles in the present study may reflect the influence of environmental deposition or abrasion-derived particles associated with repeated food-contact processes. However, particle color alone cannot be directly linked to a specific contamination source because pigments and additives used during polymer manufacturing may influence particle appearance [34]. Although no statistically significant differences in color distribution were observed between producer types, the unequal distribution of color categories may indicate selective persistence or generation of certain particle types within the production environment.

Morphological analysis demonstrated that fiber-type particles represented the dominant morphology in both producer groups, although fragment-type particles were also observed in some industrially packaged products. The predominance of fiber-type particles observed in the present study is generally consistent with previous dairy-product studies and with the findings of Akkemik et al. [22], who also reported filament-shaped particles as the dominant morphology in ice cream samples. Fiber-type contamination has frequently been associated with airborne deposition, textile-related sources, repeated handling, and abrasion of flexible food-contact materials [33]. Akkemik et al. [22] suggested that airborne contamination and repeated contact with food-contact materials may contribute to the predominance of filament-type particles in ice cream products. Similarly, the dominance of fiber-type MPs in the present study may be associated with repeated handling and environmental exposure during production and retail sale.

### 4.2. Potential Contamination Pathways and Food Safety Implications

The higher contamination frequency observed in locally produced unpackaged products should not be interpreted as definitive evidence of a single contamination source. Instead, the findings may reflect the combined influence of repeated manual handling, open-service conditions, environmental exposure, contact with processing equipment, and possible airborne deposition. Similar contamination pathways have previously been discussed in dairy-product contamination studies and food-processing environments [11]. Unlike industrially packaged products, unpackaged local ice creams are continuously exposed to repeated serving practices, ambient air, storage cabinet opening, and direct contact with food-contact surfaces. These conditions may increase the probability of environmental contamination and particle transfer during retail handling.

Although extensive contamination-control precautions and blank procedures were applied throughout the study, the possibility of airborne fiber contamination cannot be completely excluded. Procedural blanks and airborne contamination controls were evaluated throughout the analytical process, and particles corresponding to contamination profiles observed in blank controls were excluded from the final dataset. Nevertheless, because fiber-type particles dominated the analyzed samples, the potential contribution of airborne contamination should still be considered when interpreting the findings. Similar methodological limitations associated with airborne fiber contamination have previously been discussed in dairy-product and beverage-related MP studies [11,22]. In addition, because field blanks were not included during retail sampling and transportation, the relative contribution of contamination introduced during product handling, serving, transport, and the retail environment could not be distinguished from contamination potentially associated with the product itself. Therefore, the higher MP occurrence observed in locally produced unpackaged products should be interpreted with appropriate caution, as contamination may reflect the combined influence of multiple environmental and handling-related sources rather than a single contamination pathway.

The use of 30% H_2_O_2_ digestion facilitated the removal of the highly organic dairy matrix; however, oxidative digestion may potentially alter sensitive polymer surfaces or contribute to the loss of fragile particles. Therefore, the reported MP abundances should be interpreted as conservative estimates rather than absolute contamination levels. Similar limitations associated with oxidative digestion procedures have also been reported in previous food-related microplastic studies [36]. Furthermore, although the applied H_2_O_2_ digestion protocol was selected based on previously published studies investigating MPs in food matrices, spike-and-recovery experiments were not performed in the present study. Therefore, the recovery efficiency of the analytical procedure could not be quantitatively evaluated. In addition, the potential effects of oxidative digestion on particle morphology, discoloration, and polymer identification were not experimentally assessed and should therefore be considered when interpreting the findings. Future studies should include polymer-specific spike-and-recovery experiments to further validate the analytical procedure and evaluate the potential influence of oxidative digestion on particle recovery and characterization.

In addition to contamination occurrence, a deterministic exposure assessment was performed to provide a preliminary estimation of potential dietary intake associated with ice cream consumption. The calculated mean EDI values were 0.17 particles kg BW^−1^ day^−1^ for industrially packaged products and 0.36 particles kg BW^−1^ day^−1^ for locally produced unpackaged ice creams. Furthermore, the maximum EDI value observed among locally produced samples reached 0.68 particles kg BW^−1^ day^−1^. These findings suggest that consumers of locally produced unpackaged ice creams may experience higher dietary exposure to MPs than consumers of industrially packaged products. The higher exposure estimates observed for locally produced samples are consistent with the greater MP detection frequency recorded in this group.

Recently, Akkemik et al. [22] investigated microplastic contamination in commercially available ice cream products in Türkiye and demonstrated that ice cream consumption may contribute to dietary MP exposure when evaluated using a deterministic exposure model. Similarly, Başaran et al. [6] reported that dairy-product consumption may represent a potential route of human exposure to MPs. Collectively, these findings support the view that dairy-based products may contribute to overall dietary MP intake. However, unlike Akkemik et al. [22], the present study comparatively evaluated industrially packaged and locally produced unpackaged ice creams and demonstrated higher exposure estimates in locally produced products. Nevertheless, because exposure calculations were based on average consumption assumptions and only MPs detected within the investigated size range were considered, the reported values should be interpreted as preliminary estimates rather than definitive exposure levels.

However, because the pRi values used in this study represent a relative contamination indicator rather than a direct toxicological assessment, the results should not be interpreted as definitive evidence of human health risk. Although locally produced unpackaged products exhibited higher average pRi values, the limited number of locally produced samples (*n* = 5) restricts statistical power and generalizability; therefore, the observed differences should be regarded as indicative rather than conclusive. Akkemik et al. [22] similarly emphasized that the toxicological implications of food-borne MPs remain uncertain and that further studies incorporating exposure assessment and toxicological validation are required to better understand potential health risks.

The relatively limited number of locally produced unpackaged samples should also be considered when interpreting comparisons between producer types. Although statistically significant differences were observed, the present study was designed as an exploratory comparative investigation rather than a nationwide survey. Therefore, the reported contamination frequencies should be interpreted as preliminary observations rather than population-level estimates and should be confirmed using larger and more geographically representative sample sets.

Overall, the findings highlight the importance of improving hygiene practices, material selection, handling procedures, and environmental controls during production and retail, particularly for unpackaged products, to minimize potential microplastic contamination and enhance food safety.

## 5. Conclusions

This study comparatively evaluated microplastic contamination in industrially packaged and locally produced unpackaged ice cream products marketed in Türkiye. Microplastics were detected in both product types, with locally produced unpackaged ice creams exhibiting a higher detection frequency and greater mean contamination than industrially packaged products. Fiber-shaped particles predominated in locally produced samples, whereas industrially packaged products exhibited greater morphological diversity. EVA and ABS–EVA were the predominant polymer types identified. The deterministic exposure assessment indicated that consumption of locally produced unpackaged ice creams may result in higher dietary microplastic exposure than consumption of industrially packaged products, although these estimates should be considered preliminary because of the limited sample size and the exploratory nature of the study. Overall, these findings highlight the importance of improving hygiene practices, material selection, handling procedures, and environmental controls during the production and retail sale of unpackaged dairy products. Further studies involving larger and more geographically representative sample sets, comprehensive quality-control validation, and additional contamination-monitoring approaches are needed to improve our understanding of microplastic contamination and its implications for food safety.

## Figures and Tables

**Table 1:** Characteristics of industrially packaged and locally produced ice cream samples analyzed for microplastic contamination.

Code	Product Type	Brand	Packaging Status	Packaging Material	Weight (g)	MP Detection	Expiry Date
S1	Industrial	A	Packaged	PP	489	Positive	February 2027
S2	Industrial	A	Packaged	PP	49	Negative	January 2027
S3	Industrial	A	Packaged	PP	48	Positive	January 2027
S4	Industrial	A	Packaged	PP	85	Negative	February 2027
S5	Industrial	B	Packaged	PP	76	Negative	March 2027
S6	Industrial	C	Packaged	PP	45	Positive	December 2026
S7	Industrial	A	Packaged	PP	145	Positive	January 2027
S8	Industrial	B	Packaged	PP	63	Negative	March 2027
S9	Industrial	B	Packaged	PP	45	Negative	March 2027
S10	Industrial	C	Packaged	BOPP	76	Positive	May 2027
S11	Industrial	D	Packaged	PP	98	Negative	May 2027
S12	Industrial	D	Packaged	PP	68	Negative	March 2027
S13	Industrial	C	Packaged	PP	45	Negative	December 2026
S14	Industrial	E	Packaged	PP	81	Negative	April 2027
S15	Industrial	E	Packaged	PP	120	Negative	April 2027
S16	Industrial	E	Packaged	PP	60	Positive	April 2027
S17	Industrial	A	Packaged	PP	121	Positive	May 2027
S18	Industrial	A	Packaged	PP	250	Negative	May 2027
S19	Industrial	B	Packaged	PP	100	Positive	December 2026
S20	Local	F	Unpackaged	-	250	Positive	NA
S21	Local	G	Unpackaged	-	300	Positive	NA
S22	Local	H	Unpackaged	-	300	Positive	NA
S23	Local	I	Unpackaged	-	200	Positive	NA
S24	Local	J	Unpackaged	-	250	Positive	NA

NA: Not applicable. Locally produced unpackaged ice cream samples were sold without individual packaging or expiry date labeling.

**Table 2:** Estimated daily and lifetime microplastic exposure associated with ice cream consumption.

Parameter	Industrially Packaged	Local Unpackaged
Age range	15+	15+
Body weight (kg)	73.7	73.7
Ice cream consumption (g/day)	12.6	12.6
Mean EDI (particles kg BW^−1^ day^−1^)	0.17	0.36
EDI range (particles kg BW^−1^ day^−1^) *	0.17	0.17–0.68
Annual EDI (particles kg BW^−1^ year^−1^)	62.05	131.40
Annual EDI range (particles kg BW^−1^ year^−1^)	62.05–62.05	62.05–248.20

* The identical minimum and maximum EDI values observed for industrially packaged products reflect the uniform microplastic concentration detected across all positive samples in this group, resulting in a single calculable exposure estimate.

## Data Availability

The original contributions presented in this study are included in the article/Supplementary Material. Further inquiries can be directed to the corresponding author.

## Supplementary Materials

The following supporting information can be downloaded at https://www.mdpi.com/article/10.3390/foods15142517/s1: Figure S1: Representative microscopic images and corresponding ATR-FTIR spectra of the polymer types (PE, EVA, PET, and ABS–EVA) identified in the analyzed ice cream samples; Table S1: Results of procedural blank analyses performed during microplastic extraction and ATR-FTIR verification; Table S2: Classification of visually suspected particles following ATR-FTIR verification.
