# Supplementary material for: Microplastic Contamination in Industrially Packaged and Locally Produced Ice Creams: Occurrence, Characteristics, Exposure Assessment, and Pollution Risk
Source: Foods. 2026 Jul 16;15(14):2517. doi: 10.3390/foods15142517 (PMC13409447; doi:10.3390/foods15142517)
Supplement: Supplementary file 1 [file foods-15-02517-s001.zip › foods-4414292-supplementary.pdf]

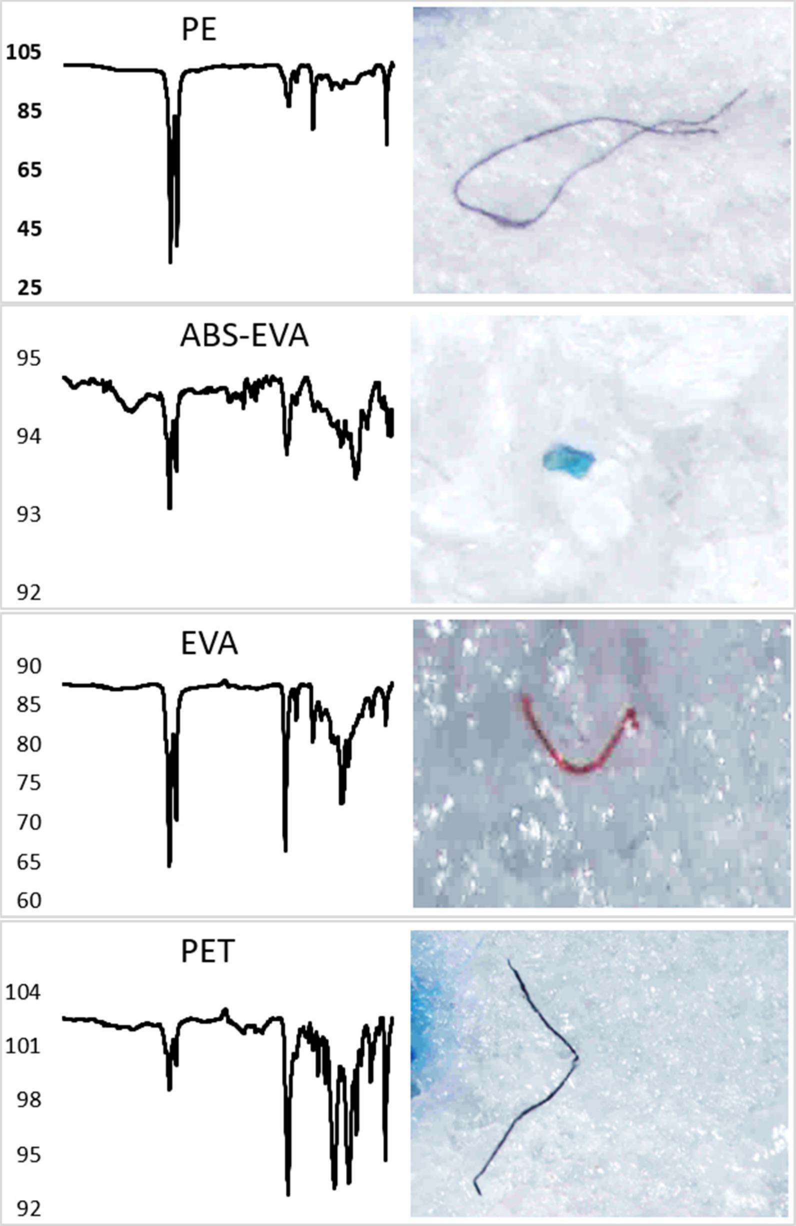

Figure S1. Representative microscopic images and corresponding ATR-FTIR spectra of the polymer types (PE, EVA, PET, and ABS–EVA) identified in the analyzed ice cream samples.

Table S1. Results of procedural blank analyses performed during microplastic extraction and ATR-FTIR verification.

Procedural blank	No. of particles	Morphology	Color	Polymer (ATR-FTIR)	Action taken
PB1	0	–	–	–	–
PB2	1	Fiber	Green	Polyester	Corresponding particle excluded from the final dataset
PB3	0	–	–	–	–

Table S2. Classification of visually suspected particles following ATR-FTIR verification.

Classification after ATR-FTIR	Number of particles
Confirmed microplastics	21
Cellulose-based particles	13
Hair	2
Ambiguous / unidentified	2
**Total visually suspected particles**	**38**
